# The role of PMCT for the assessment of the cause of death in natural disaster (landslide and flood): a Sicilian experience

**DOI:** 10.1007/s00414-021-02683-z

**Published:** 2021-09-02

**Authors:** Cristina Mondello, Gennaro Baldino, Antonio Bottari, Daniela Sapienza, Fabrizio Perri, Antonina Argo, Alessio Asmundo, Elvira Ventura Spagnolo

**Affiliations:** 1 Department of Biomedical and Dental Sciences and Morphofunctional Imaging, University of Messina, via Consolare Valeria, 1, 98125 Messina, Italy; 2 Section of Legal Medicine, Department of Health Promotion Sciences, Maternal and Infant Care, Internal Medicine and Medical Specialties (PROMISE), University of Palermo, Via del Vespro, 129, 90127 Palermo, Italy

**Keywords:** Post-mortem CT, Natural disaster, Forensic sciences, Cause of death, Traumatic injury, Asphyxia

## Abstract

In this report, the authors provide a contribution of PMCT in assessing the cause of death due to natural disasters. Here, the PMCT findings of 43 subjects who died during both landslide and flood were described. The post-mortem imaging revealed, clearly, traumatic injuries and/or the presence of foreign material in airways allowing to assess the cause of death of each subject, together with external inspection and the collected circumstantial data. Particularly, the PMCT has been helpful for characterization and localization of the clogging substance in airways providing findings on bronchial branches involvement. Moreover, the investigation offered detailed data on skeletal injuries in all anatomic districts and put in evidence both the precise fracturing site and the characteristics of fracture stubs for each bone fracture. This report supports the recommendation of the virtual autopsy in a case with several victims, as in natural disasters, and its role as an alternative diagnostic investigation when the standard autopsy is not feasible.

## Introduction

Italy is a country with a high risk of landslides and flood hazards [1]. The substantial enlargement of urbanized areas without correct territorial planning led to a considerable increase of risk factors exposing to landslides and floods. In fact, artificial surfaces have passed from 2.7% in the 1950s to 7.65% in 2017 [2]. Moreover, the mountain and hilly rural areas’ abandonment determined, also, the lack of supervision and management of the territory. Sicily is an Italian region with a moderate risk of landslides and floods, and it was reported the high concentration of population at risk living in high hazard zones [2].

The rapid phenomena occurring in some such cases (rockfall, mud, and debris flow or land covered by water), characterized by high velocity and high destructive power, can involve towns, causing destruction and life losses. In these cases, the victims can report several and complex injuries requiring a comprehensive forensic approach in which post-mortem computed tomography (PMCT) represents a fundamental tool providing valuable findings.

PMCT is widely used in the forensic field for several purposes, mainly assessing the cause of death, individual identification, and estimating the post-mortem interval [3–7]. Moreover, it has great relevance for analysis (detection, visualization, and practical description) of traumatic injuries [8], leading to considerable clout in assessing traumatic death.

The present paper reports the PMCT findings in victims of landslide and flood occurred in Sicily. This work aimed to show the usefulness of PMCT to analyze injuries associated with these two kinds of natural disasters and describe the related differences on which the death mechanisms of each subject were determined.

## Presentation of the cases

On October 1st, 2009, in two small villages of Messina Province (in the north-eastern Sicily), already considered to be at high hydrogeological risk, a violent storm (up to 220/230 mm of rainfall over 3–4 h) caused the river overflow and landslides, determining a flow of mud and debris that swept away building and dwellings. The storm caused 33 victims (group 1) of the flood, respectively 16 females (aging between 28 and 80 years; median age 54 years), 14 males (aging between 22 and 86 years; medium age 55), 3 children (respectively a 2 years old male and a 6-year-old male), and a body remains belonging from one subject.

On 2018, November 3rd, a vast flood fell in the Palermo neighbors (Altavilla Milicia), mainly interesting the seaside. In a small town near Palermo, rain fell particularly hard, and a little river overflowed, carrying down a large amount of mud and debris. This wave of water mixed with mud and debris overwhelmed a house in which 9 people died. The victims (group 2) were 4 females (aging between 34 and 65 years; median age 50 years), 3 males (aging between 15 and 69 years; medium age 39), and 2 children (respectively 3-year-old male and 1-year-old male). The flood engulfed another subject while he was in the car.

In both cases, the judicial authorities requested the forensic examination to establish the causes of death for each victim and the compatibility of injuries with the natural event.

## Methods

The PMCT was performed on 42 bodies and body remains belonging from one subject in sealed body bags in the supine position. The investigation on group 1 (estimated PMI between 36 and 60 h) was carried out with a 64-row dual-energy scanner (Somatom Definition, Siemens, Erlangen, Germany) using the following scanning parameters: slice thickness of 1 mm, interval of reconstruction of 1 mm, tube voltage of Kv 140, and mAs between 200 and 350, according to the body mass index of the corpses. Reconstructions were made using standard and bone filters. Post-processing was performed by a multimodality workstation (MMWP, Siemens). Then, the external examination of each body was provided.

The investigation on group 2 (estimated PMI between 24 and 48 h) was performed using PMCT with a 128-slice MDCT scanner (Somatom Definition AS®, Siemens Healthcare, Erlangen, Germany): tube voltage of 120kVp, with an effective tube current of 120–160 effective mAs; gantry rotation time of 0.5 s, beam pitch of 1.2, and table speed of 46 mm per gantry rotation; overlapped slices with a thickness of 0.6 mm. Reconstructions were made using standard and bone filters. Post-processing was performed by a multimodality workstation (Singovia® Siemens Healthcare Erlangen Germany). Then, the external examination of each body was provided.

The Hounsfield unit (HU) was evaluated in cases showing foreign material in airways at the preliminary image analysis to characterize the density; particularly, the HU median value was calculated combining five areas at the trachea, bilateral main bronchi, and bilateral lung/bronchia in individual cases.

## Results

The PMCT data observed in each body are summarized in Tables 1 and 2.

**Table 1:** Summary of the PMCT data observed in each body belonged from group 1, together with the cause of death

*N*	Sex	Age	CT findings	Cause of death
1	F	47	Foreign body in the upper airways; small foreign bodies in bronchi; foreign body in esophagus; humerus fracture; ribs fractures; atlas-epistropheus dislocation	Asphyxia due to airway occlusion by solid material with polytrauma
2	F	80	Dense foreign material in airways, esophagus and stomach	Asphyxia due to airway occlusion from dense material aspiration
3	F	71	Dense foreign material in airways, esophagus and stomach	Asphyxia due to airway occlusion from dense material aspiration
4	F	42	Dense foreign material in airways, esophagus and stomach; pelvis fracture; ulna fracture; right leg fracture	Asphyxia due to airway occlusion from dense material aspiration with polytrauma
5	M	26	Skull fracture; humerus fracture; rib fractures; right femur, tibia and fibula fracture	Polytrauma
6	F	33	Right zygomatic fracture; multiple rib fractures; D12 fracture with retrolisthesis	Traumatic shock
7	M	86	Dense foreign material in airways and esophagus; multiple rib fractures; radio and ulna fractures; D4 and D10 fractures; left and right iliac wing fractures; right femur fracture; right tibia and fibula fractures	Asphyxia due to airway occlusion from dense material aspiration with polytrauma
8	M	22	Right upper limb amputation; right tibia and fibula fractures with partial amputation	Traumatic shock
9	M	27	Multiple rib fractures; left lung collapse	Compression asphyxia
10	F	44	Dense foreign material in esophagus	Restraint asphyxia
11	M	50	Multiple skull fractures; maxillary fracture; multiple rib fractures	Polytrauma
12	M	76	Right femur fracture; left femur, tibia and fibula fractures	Traumatic shock
13	F	48	Dense foreign material in airways and esophagus	Asphyxia due to airway occlusion from dense material aspiration
14	M	73	Dense foreign material in esophagus and stomach; multiple anterior and posterior rib fractures; pelvis fractures; left tibia fracture	Compression asphyxia with polytrauma
15	M	72	Dense foreign material in esophagus	Restraint asphyxia
16	M	28	Decapitation at C4-C5 level; D3-D4 fracture and disarticulation; ribcage collapse and destruction; left upper limb amputation	Polytrauma with decapitation due to explosion
17	M	64	Jaw fracture; sternum and multiple rib fractures with lungs and heart damage; right scapula and clavicle fractures; multiple pelvis fractures; right tibia and fibula fractures	Polytrauma
18	M	74	Multiple rib fractures with lung collapse	Compression asphyxia
19	F	40	Multiple rib fractures with lung collapse	Compression asphyxia
20	F	32	Dense foreign material in upper airways, esophagus and stomach; left temporo-zygomatic fracture; jaw fracture; multiple rib fractures	Asphyxia due to airway occlusion from dense material aspiration with polytrauma
21	F	75	Dense foreign material in airways and esophagus; multiple rib fractures; pelvis fractures	Asphyxia due to airway occlusion from dense material aspiration with polytrauma
22	M	46	Maxillo-facial fractures; dense foreign material in upper airways and esophagus; multiple rib fractures; right clavicle fracture; left ischio-pubic branch fracture	Asphyxia due to airway occlusion from dense material aspiration with polytrauma
23	F	78	Skull fractures; maxillo-facial fractures; wide penetrating wound at thorax and abdomen; vertebral column disarticulation at D3-D4 level; pelvis fractures; left tibia and fibula fractures	Polytrauma
24	F	43	(Body parts: pelvis and lower limbs) left and right tibia and fibula fractures	Traumatic shock
25	M	36	Fractures of all ribs; D3 burst fractures with disarticulation	Compression asphyxia
26	F	59	Multiple anterior and posterior rib fractures; D7 fracture with disarticulation; pelvis fracture; right lower limb amputation at mid-thigh level; left femorus fracture	Compression asphyxia with polytrauma
27	F	67	Sternum fracture; D12 fractures with disarticulation	Traumatic shock
28	F	63	Dense foreign material in upper airways	Asphyxia due to airway occlusion from dense material aspiration
29	M	2	Skull fractures; dense foreign material in airways, esophagus and stomach; right ilio-pubic fracture; right and left femorus fractures	Asphyxia due to airway occlusion from dense material aspiration with polytrauma
30	M	6	Cervical spine disarticulation at C3-C4 level; vertebral column disarticulation at D12-L1 level; right ilio-pubic and ischio pubic branch fractures; right femorus fractures; right tibia and fibula fractures	Polytrauma
31	F	28	Dense foreign material in airways, esophagus and stomach	Asphyxia due to airway occlusion from dense material aspiration
32	M	67	Foreign material in upper airways, esophagus and stomach; partial amputation of left foot at ankle level	Asphyxia due to airway occlusion from dense material aspiration
33	M	10	Foreign material in upper airways, esophagus and stomach	Asphyxia due to airway occlusion from dense material aspiration

**Table 2:** Main PMCT findings and cause of death of the group 2 subjects

*N*	Sex	Age	TC finding	Cause of death
1	M	69	Low-density fluid in paranasal sinuses, larynx and pharynx; low density fluid mixed with hyperdense material in trachea and bronchi; lung consolidations in hilar site; low density fluid mixed with hyperdense material in esophagus and stomach	Asphyxia due to airway occlusion from fluid/dense material aspiration
2	F	62		
3	F	40		
4	M	15		
5	M	33		
6	F	34		
7	F	65		
8	M	3		
9	M	44		
10	M	1	Low-density fluid in paranasal sinuses; low density fluid mixed with hyperdense material in larynx and pharynx; lung consolidations in hilar site; low density fluid mixed with hyperdense material in esophagus and stomach	

**Fig. 1:**
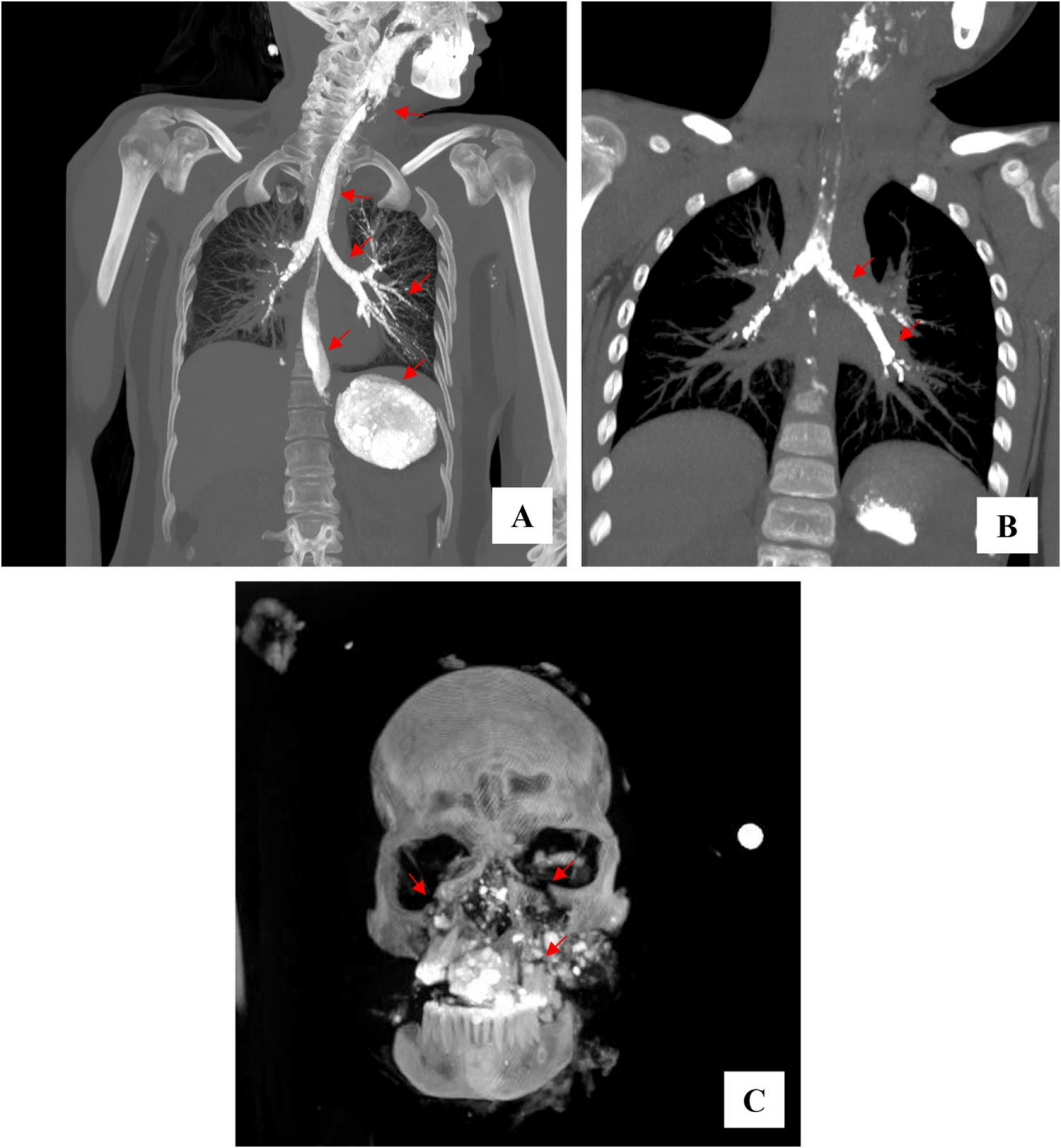
PMCT images related to subjects died during Messina flooding (group 1). Coronal view of MIP (**A**) and MPR (**B**) reconstructions showing hyperdense material in both upper and lower airways, filling up to segmental bronchi, and in esophagus and stomach because of ingestion and inhalation (arrows). (**C**) Coronal view of VR reconstruction of the skull with multiple maxillo-facial fractures (arrows) caused by impact with rubble

**Fig. 2:**
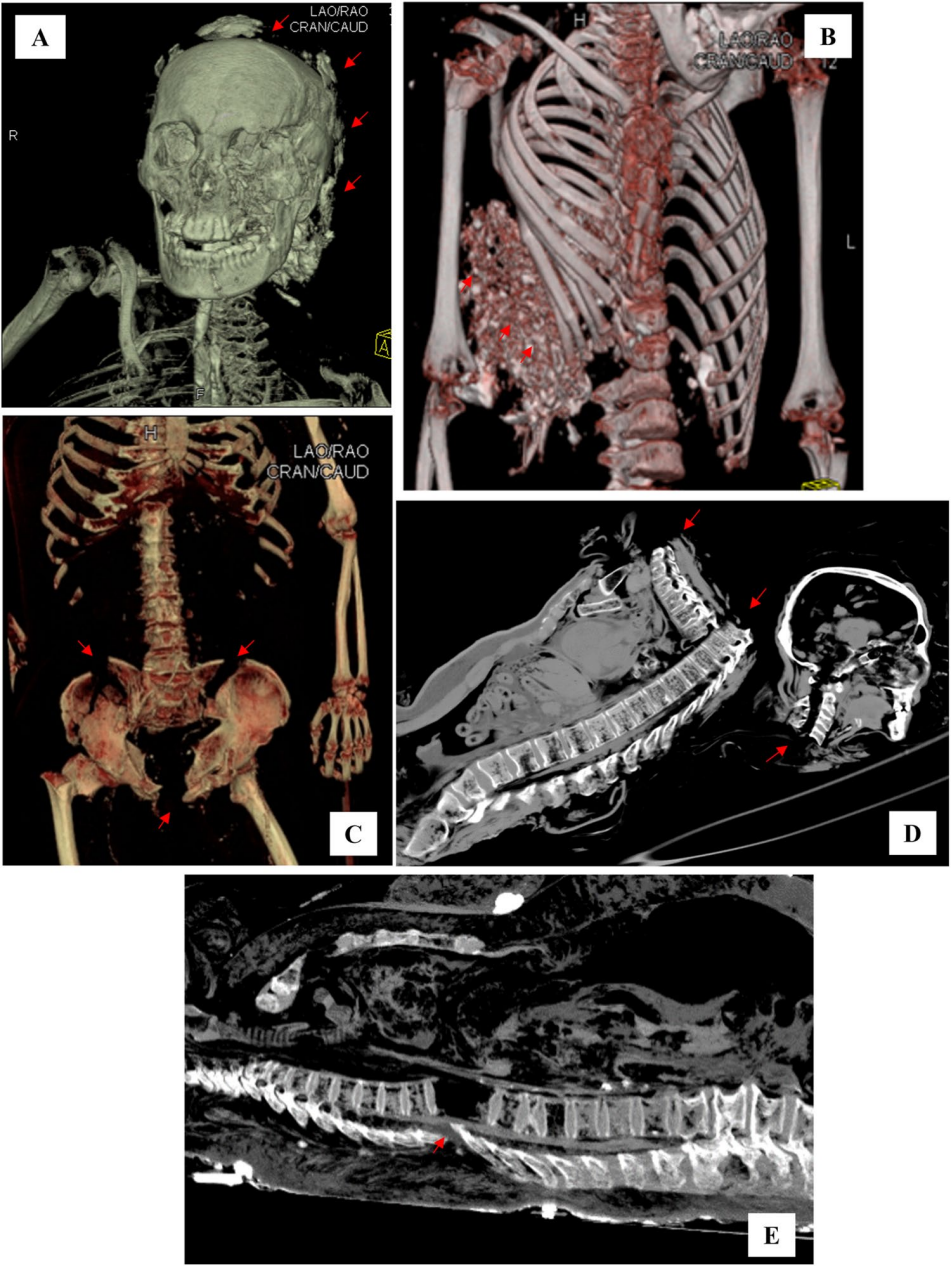
PMCT images related to subjects died during Messina flooding (group 1). (**A**, **B**, **C**) VR reconstructions on paracoronal planes showing traumatic injures (arrows) determined by landslide on skull (**A**), rib cage (**B**), with rubble penetrating into thorax cavity, and pelvis (**C**). (**D**, **E**) MPR reconstructions on sagittal view demonstrating high-grade lesions (arrows) with decapitation as dramatic effects of a blast

In group 1, the following main findings were observed: dense foreign material in airways in 14 cases (Fig. 1); multiple rib fractures with lung collapse in 6 cases; traumatic injuries (i.e., limb amputation, vertebral column lesions, upper and/or lower limb fractures, pelvis fractures, and maxilla-facial fractures) in 15 cases (Fig. 2), one of which showed decapitation. In 7 cases, the coexistence of foreign material in airways and polytrauma was highlighted. The external examination revealed severe traumatic injuries, as detected by the PMCT, and skin injuries (i.e., contusions, abrasions, and lacerations); body no. 16 showed extensive carbonization.

The PMCT investigation, together with both body inspection and circumstantial data about the body discovery sites, allowed to define the cause of death as acute asphyxia in 20 cases: in 14 cases, the asphyxiated mechanism was the occlusion of the airway by foreign material (airway clogging); in 5 cases, with rib fractures with or without lung damage, the mechanism was the compression asphyxia; in 2 cases, without both PMCT findings of airway occlusion and traumatic lesions, the restraint asphyxia was considered the probable mechanism. In 12 victims, the identified cause of death was polytrauma due to crushing or blunt force injuries; in one case (no. 16), the observed high-grade lesions, also considering the circumstantial data, were related to a blast.

**Fig. 3:**
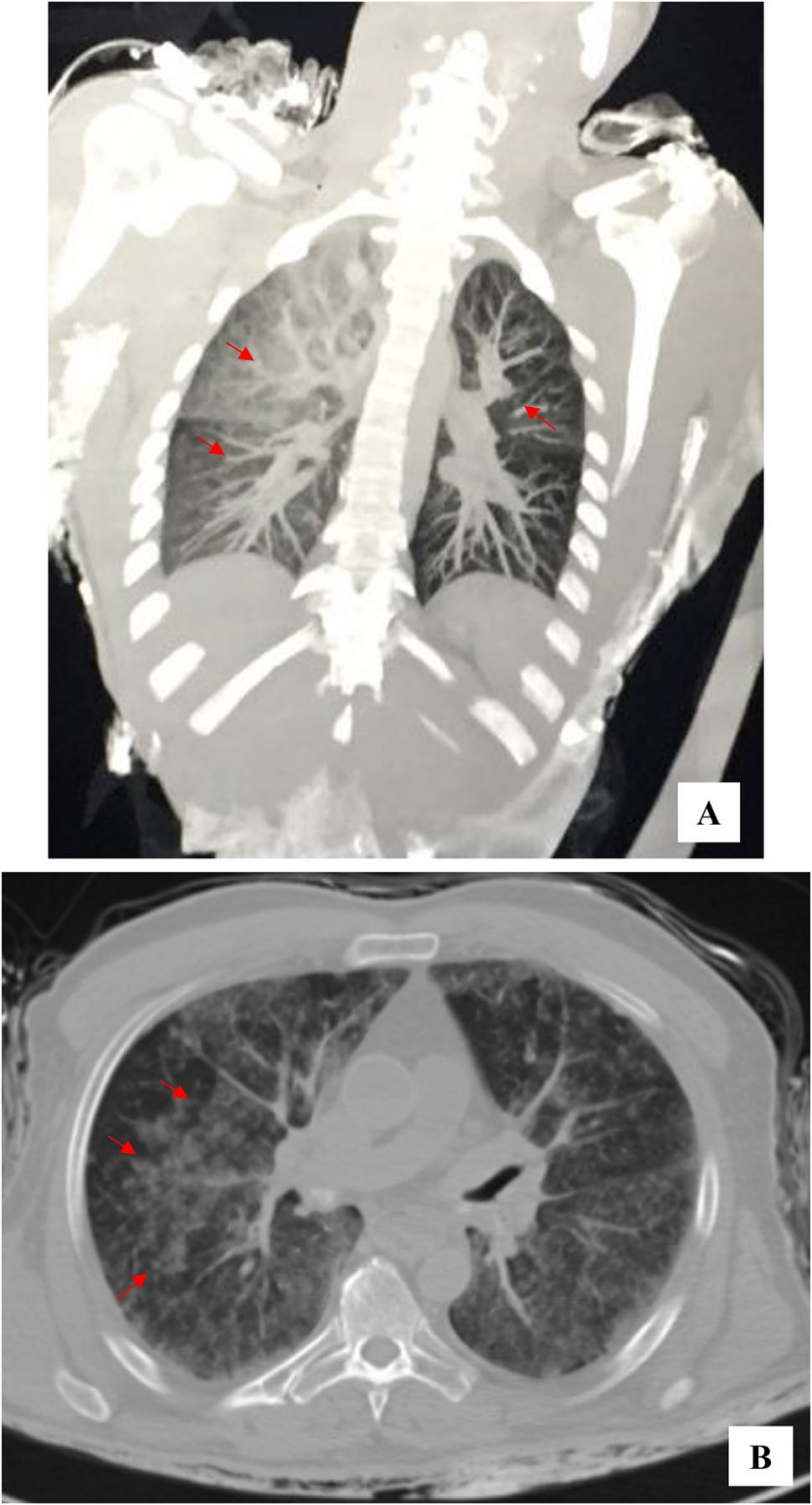
PMCT images related to subject died during Palermo flooding (group 2). MPR reconstruction on coronal (**A**) and axial (**B**) planes showing several areas of ground-glass attenuation (arrows) of the lungs caused by inhalation of predominantly fluid material

Regarding group 2, PMCT revealed, as a single finding, the presence of low dense foreign material clogging the airways in all bodies (Fig. 3); at body inspection, foam around the mouth was observed in each case and no traumatic injuries were found. The cause of death was defined as acute asphyxia due to airway occlusion.

The Hounsfield unit (HU) analysis performed in subjects who died from acute asphyxia due to airway occlusion and belonged from both groups (Table 3) revealed a higher value in group 1, demonstrating a greater density of the clogging material compared to those of group 2.

**Table 3:** Summary of the median value of HU detected in each subject with foreign material in airways

Group 1				Group 2			
*N*	Sex	Age	HU	*N*	Sex	Age	HU
1	F	47	740.69	1	M	69	300
2	F	80	781.0	2	F	62	300
3	F	71	739.82	3	F	40	160
4	F	42	742.3	4	M	15	270
7	M	86	748.83	5	M	33	260
13	F	48	793.56	6	F	34	190
20	F	32	740.23	7	F	65	300
21	F	75	754.37	8	M	3	164
22	M	46	738.21	9	M	44	210
28	F	63	775.92	10	M	1	250
29	M	2	794.65				
31	F	28	764.12				
32	M	67	782.0				
33	M	10	749.17				

## Discussion

PMCT applications focus mainly on the analysis of non-natural death as cases related to a crime carried out by firearms, knives, and blunt instruments [9, 10]. Moreover, several literature reports highlight the fundamental role of PMCT in traumatic events involving several persons as explosions, air and rail disasters, and shipwrecks [11–13]. The main advantages are the ability to provide rapid and complete results on the main parenchymal changes, bone lesions, and the presence of foreign bodies. In this way, PMCT is very useful as a preliminary investigation in the forensic setting, being a strategic guide during autopsies. The scanning process takes only a few minutes, and, following the image processing, the results are available almost immediately. Other advantages are the digital re-use of acquired data and the possibility of re-analyzing the images many times.

Some researchers suggest that PMCT might be an alternative to the autopsy providing data for the definitive assessment of the causes of death. In fact, PMCT can be used as the primary investigation tool demonstrating the cause of death, especially in traumatic events with several victims, because it offers quick information on the lesion type and distribution [14]. Cirelli et al. [15] tested the PMCT efficacy in traumatic death, comparing to autopsy findings, reporting accuracy 84%, sensitivity 82%, specificity 86%, positive predictive value 90%, and negative predictive value 86%.

This evidence supports the PMCT in natural disasters such as floods, earthquakes, and tsunami, in which death is generally due to moderate or high-grade traumatic injuries.

In this paper, the authors report the PMCT images’ contribution to assessing the cause of death in subjects who died during a landslide and a flood. The investigation provided very detailed data on skeletal injuries in all anatomic districts allowing to describe for each bone fracture both the precise fracturing site and the characteristics of fracture stubs (i.e., compound/comminuted fractures). The imaging allowed the description of bone lesions in the facial skeleton, pelvis, and extremities that are considered areas not easily accessible for dissection [16]. Skeletal chest findings and, particularly, rib fracture location and characteristics concurred (together with circumstantial data) to explain the traumatic mechanism (i.e., crushing trauma); several authors reported the strong agreement between PMCT and autopsy in the evaluation of rib fractures [17, 18]. Clear findings were also observed on the spine, confirming the usefulness of imaging to detect both fractures and dislocation [16, 19]. Other interesting data were found in the airway examination, in which the post-mortem imaging highlighted the presence of foreign material with different densities in both upper and lower tracts. Comparing the subjects who died during the two natural phenomena, clogging material had a lower density in group 2, in which it was observed up to the smallest segmental bronchi; in group 1, the foreign material was predominantly found up to the lobar bronchi. Thus, PMCT has served to characterize and localize the clogging substance in airways, providing useful findings on bronchial branch involvement and, consequently, on the asphyxial death [20]. Remarkable were also the HU value differences observed between the two groups, demonstrating a greater density of foreign airway material in landslide cases if compared to those of flooding.

It must be highlighted the importance, in such cases, to consider the theoretical possibility of passive post-mortem penetration of foreign bodies in the airways in the definition of the cause of death. By similarity, this condition is largely analyzed in drowning, and several studies were performed to provide methods and markers allowing to distinguish between drowning and submersion of a corpse (i.e., diatoms analysis) [21, 22]. Obviously, the post-mortem penetration of water differs from post-mortem penetration of foreign material (dense or solid) because of different physic characteristics influencing the ability to penetrate passively in a system as the airways; this consideration is supported by the different airways’ localization of foreign material (with different density) in the two described groups. Thus, it can be supposed that dense foreign material, as observed in the present study, encounters greater resistance to passive penetration into the respiratory tract than water resulting in a more superior airway localization. It follows that the presence of the dense foreign material in the smallest bronchi, as observed in the present report, may suggest an “active” penetration related to subject respiration.

The present analysis revealed the limitation of PMCT sensibility in skin injury evaluation. The CT allowed the detection of severe integumentary lesions (i.e., case 23), but a better analysis of their characteristics was provided by external examination. Not detectable by PMCT were contusions and excoriations.

In conclusion, this report supports the recommendation of virtual autopsy as an alternative diagnostic activity if a standard autopsy is not possible. PMCT is very useful in cases with several victims, as natural disasters, appearing to be a screening test for traumatic deaths providing equal or superior skeletal findings to autopsy. Moreover, based on the findings here described, PMCT imaging can be recommended for detecting foreign material in airways, allowing a good evaluation of both density and localization of the clogging substance.
